# Comparison of blood pool and extracellular gadolinium contrast for functional MR evaluation of vascular thoracic outlet syndrome

**DOI:** 10.1186/1532-429X-15-S1-E81

**Published:** 2013-01-30

**Authors:** Ruth P Lim, Mary T Bruno, Andrew B Rosenkrantz, Danny C Kim, Jane Kwon, Amy Sabach, Amy Pastva, William F Chaplin, Mark Butler, Thomas P Mulholland, Olugbenga Ogedegbe

**Affiliations:** 1 Radiology, Austin Health, Heidelberg, VIC, Australia; 2 Radiology, NYU Langone Medical Center, New York, NY, USA; 3 Clinical Translational Sciences Institute, NYU Langone Medical Center, New York, NY, USA; 4 Department of Psychology, St John's University, Jamaica-Queens, NY, USA

## Background

MR angiography (MRA) is useful for vascular thoracic outlet syndrome (TOS) assessment. With standard extracellular contrast (ECA), two injections are administered in arm abduction and then adduction, with relatively high total dose. Our purpose was to compare TOS MRA image quality, vessel contrast, and detection of vascular pathology between single-injection low-dose blood pool agent (BPA) using gadofosveset trisodium, and dual-injection ECA using gadopentetate dimeglumine.

## Methods

31 patients (21 F, mean 36.5 years) with suspected vascular TOS underwent BPA (n=18) or ECA MRA (n=13) at 1.5T. T1 weighted 3D spoiled gradient echo imaging over 4 time points (abduction-early, abduction-late, adduction-early, adduction-late) was performed with injection via the less symptomatic arm. For BPA, a 0.03 mmol/kg dose was given in abduction only. For ECA, 0.075 mmol/kg was injected in abduction then adduction (total 0.15 mmol/kg). Two radiologists (R1, R2) independently evaluated images for image quality (1=non-diagnostic, 3=diagnostic, 5=excellent) and vessel contrast (1=same as muscle, 4=much brighter than muscle), with arterial contrast assessed for 1st and 3rd, and venous contrast for 2nd and 4th time points. Scores were compared with independent samples t-tests. Vascular pathology assessment was compared to reference evaluation by an unblinded experienced vascular radiologist.

## Results

For the symptomatic arm, mean image quality was diagnostic or better, and mean vessel contrast was at least moderately brighter than muscle for all time points for both BPA and ECA (Table 1). There was no significant difference between agents at abduction-early or adduction-late. There was superior venous contrast for BPA at abduction-late. At adduction-early, ECA image quality and arterial contrast were superior to BPA.

**Table 1:** Comparison of Image Quality and Vessel Contrast Scores for each time point between BPA MRA (gadofosveset trisodium) and ECA MRA (gadopentetate dimeglumine) for the symptomatic arm

Time Point	Contrast	Image Quality	p-value	Vessel Contrast	p-value
1. Abduction-early	BPA	4.58±0.39	0.92	3.97±0.12	0.57
				
	ECA	4.50±0.41		3.92±0.19	

2. Abduction-late	BPA	4.36±0.38	0.06	3.97±0.12	0.007
				
	ECA	4.12±0.30		3.73±0.26	

3. Adduction-early	BPA	3.89±0.65	0.014	3.42±0.52	<0.001
				
	ECA	4.42±0.11		3.96±0.14	

4. Adduction-late	BPA	4.17±0.49	0.09	3.86±0.29	0.18
				
	ECA	3.77±0.78		3.62±0.68	

Image Quality Scores: 1=non-diagnostic, 3=diagnostic, 5=excellent Vessel Contrast Scores: 1=same as muscle, 4=much brighter than muscle. Note arterial contrast was assessed at Time Points 1 and 3, and venous contrast at Time Points 2 and 4

**Figure 1:**
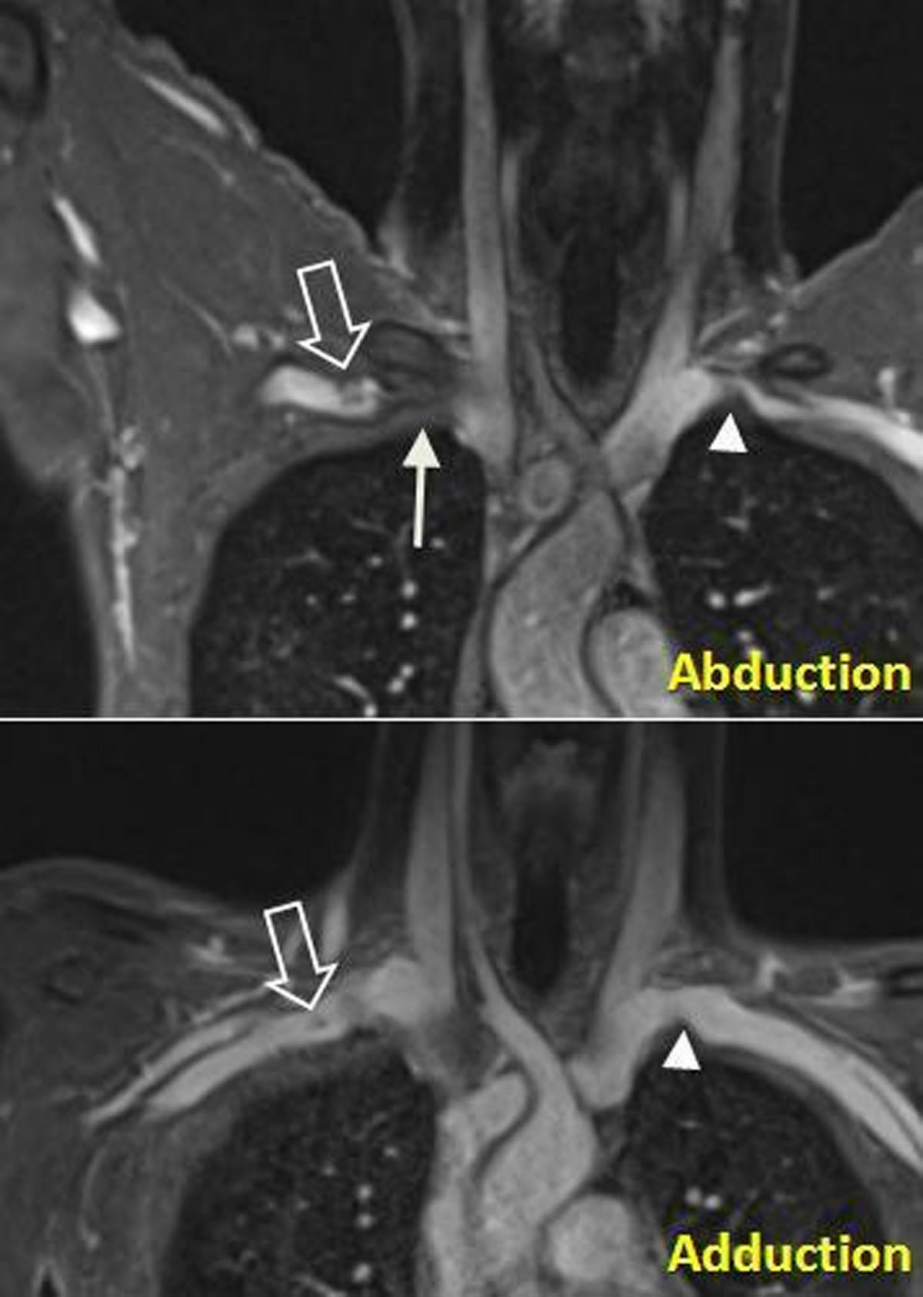
BPA-MRA images for a 24 year old male with history of right subclavian thrombosis referred for evaluation for TOS. Marked narrowing of the right subclavian vein is evident at abduction-late (solid arrow) with thrombus within the second part of the vein (open arrow). Narrowing resolves in adduction-late. Reversible narrowing of the left subclavian vein is also present (arrowhead).

For BPA, there were 3 significant subclavian artery stenoses in 3/36 arms, all identified by R1 and 1/3 by R2. 1 subclavian artery aneurysm was identified correctly by both readers. There were 20 significant venous stenoses identified in 36 arms; R1 identified 19/20 and R2 correctly identified all venous stenoses, with one false positive stenosis. 3 venous thromboses were all correctly identified by R2 and 2/3 by R1 (Figure 1).

For ECA, there were 3 arterial stenoses. All were correctly identified by R1, and 2/3 by R2. 13 venous stenoses were present at the reference standard. R1 identified 12/13 stenoses and R2 11/13, with 4 false positive stenoses.

## Conclusions

Single-injection low-dose BPA for functional MRA of vascular TOS allows similar image quality, vessel contrast, and identification of both arterial and venous pathology as standard dual-injection ECA.

## Funding

No funding to disclose.

